# Gene expression patterns of *Salpa thompsoni* reveal remarkable differences in metabolism and reproduction near the Antarctic Polar Front

**DOI:** 10.1098/rsbl.2023.0274

**Published:** 2023-12-06

**Authors:** Svenja J. Müller, Evgeny A. Pakhomov, Ilenia Urso, Gabriele Sales, Cristiano De Pittà, Katharina Michael, Bettina Meyer

**Affiliations:** ^1^ Institute for Chemistry and Biology of the Marine Environment, Carl von Ossietzky University of Oldenburg, Oldenburg, Germany; ^2^ Scientific Division Polar Biological Oceanography, Alfred Wegener Institute Helmholtz Centre for Polar and Marine Research, Bremerhaven, Germany; ^3^ Department of Earth, Ocean and Atmospheric Sciences, University of British Columbia, Vancouver, BC, Canada; ^4^ Institute for the Oceans and Fisheries, University of British Columbia, Vancouver, BC, Canada; ^5^ Department of Biology, University of Padova, Padua, Italy; ^6^ Helmholtz Institute for Functional Marine Biodiversity (HIFMB), University of Oldenburg, Oldenburg, Germany

**Keywords:** *Salpa thompsoni*, salps, reproduction, metabolism, environmental conditions, differential gene expression analysis

## Abstract

*Salpa thompsoni* is an important grazer in the Southern Ocean and most abundant in the Antarctic Polar Front (APF) region. During recent decades, their distribution expanded southwards. However, it is unclear whether salps can maintain their populations in the high Antarctic regions throughout the year owing to a poor understanding of their physiological responses to changing environmental conditions. We examined gene expression signatures of salps collected in two geographically close regions south of the APF that differed in water mass composition and productivity. The observed differences in the expression of genes related to reproductive, cellular and metabolic processes reflect variations in water temperature and food supply between the two regions studied here. Our study contributes to a better understanding of the physiological responses of *S. thompsoni* to changing environmental conditions, and how the species may adapt to a changing environment through potential geographical population shifts under future climate change scenarios.

## 1. Introduction

In the Southern Ocean, *Salpa thompsoni* occurs from the Subtropical Convergence to high Antarctic coastal seas, being most abundant in the Antarctic Polar Front region (APF) [1–3]. Salps are very efficient filter feeders, capable of exerting a grazing pressure that regionally exceeds the total primary production [4]. Furthermore, they are known to contribute to vertical carbon flux by creating fast sinking faecal pellets. However, the contribution of salps to carbon export into deeper water layers is still uncertain, because of increased retention of faecal pellets in the upper (approx. 200 m) water layers owing to a higher fragmentation rate [5,6].

During past decades, the distribution of *S. thompsoni* has shifted southwards together with a proposed decline in Antarctic krill (*Euphausia superba*) in those regions [7]. The reasons for this observed shift are not fully understood, but studies suggest ongoing ocean warming and sea ice decline as the major drivers [2,8,9]. However, the ability of salps to maintain their populations in high Antarctic regions year-round remains questionable, as successful reproduction and development may be restricted to warmer areas (greater than 1°C) [9–11]. Nevertheless, salp populations were recorded occasionally in cold waters [12,13], suggesting an ability to also reproduce under more adverse conditions. To fully understand the conditions affecting salp development and reproduction, it is therefore critical to gain additional knowledge of their physiological responses to changing environmental conditions.

To date, inter-basin variability with respect to environmental conditions was only rarely studied [13]. The two sites investigated here, although geographically close (approx. 1250 km apart), were characterized by different environmental conditions, Salpastan station (cold/unproductive) and station 10°E (warm/productive) [13,14]. The present study is based on *de novo* transcriptomic data published by Müller *et al.* [15] covering several seasons and forms (oozoids and blastozooids). Using these data, we aimed to examine the physiological responses to regional environmental conditions in the vicinity of the APF in the Atlantic Sector of the Southern Ocean during summer 2012. In particular, we focused on differences in gene expression levels between (i) forms (oozoids versus blastozooids) in the cold and unproductive environmental setting (Salpastan), and (ii) oozoids from the two study sites with contrasting environmental conditions (Salpastan versus 10°E). This will contribute to an understanding if and how physiological mechanisms may allow salps to increasingly occur in areas formerly dominated by Antarctic krill.

## 2. Material and methods

### (a) Field sampling

**Figure 1.**
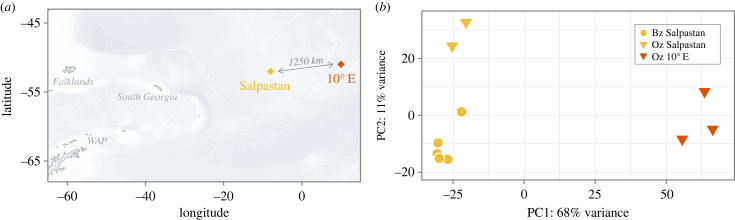
(*a*) Map of sampling stations (Salpastan, 10°E) in the APF region and (*b*) principal component analysis (PCA) of variance- stabilized gene expression levels covering samples analysed from Salpastan (*n* = 7) and station 10°E (*n* = 3). Different colours (yellow = Salpastan, red = 10°E) indicate the different sampling stations, while different shapes indicate different reproductive forms (triangle = oozoids (Oz), circle = blastozooids (Bz)).

Samples of *S. thompsoni* considered for differential gene and functional analysis were collected onboard RV *Polarstern* during ANTXXVIII/3 using a Bongo net/multiple rectangular midwater trawl from the top 450 m in the vicinity of the APF during summer 2012 (electronic supplementary material, table S1). Analysed samples included blastozooids (*n* = 5) and oozoids (*n* = 2) from the Salpastan (−52.0018, −8.00417) and oozoids (*n* = 3) from the 10°E (−50.9867, 10.0185) station (figure 1*a*; electronic supplementary material, table S1). The stomach and embryo were removed prior to extraction (see Müller *et al.* [15] for further sampling and extraction details). Environmental data were collected using a conductivity, temperature, depth (CTD) sonde (Sea- Bird Scientific SBE 911plus) and obtained from https://doi.org/10.1594/PANGAEA.840334 [14]. Raw fluorescence data were converted to chlorophyll *a* (Chl *a*) concentration using the following equation: *y* = 0.075 + 1.42*x* (*n* = 53, *R*^2^ = 0.91, *p* < 0.001) (electronic supplementary material, figures S1 and S2). This linear regression was derived by relating the fluorescence data to Chl *a* concentration from 10 to 200 m depth, which were measured using high performance liquid chromatography during the same cruise [16].

### (b) Sequence data and *de novo* transcriptome source

Seasonal- and form-specific sequence data of *S. thompsoni* published by Müller *et al.* [15] (Bioproject accession number PRJNA822688) and Batta-Lona *et al.* [17] (Bioproject accession number PRJNA279245) were used to generate the *de novo* transcriptome assembly and annotation as described in detail in Müller *et al.* [15].

### (c) Differential gene expression analysis and gene ontology enrichment analysis

Normalization of gene expression values and differential gene expression (DGE) analysis was conducted by the Bioconductor R package DESeq2 *v.* 1.32.0 [18]. Principal component analysis (PCA) was performed to estimate variation within and between groups using normalized and variance stabilized gene expression data via the *vst* transformation implemented in DESeq2. We identified differentially expressed genes (DEG) between forms (oozoids versus blastozooids) at Salpastan station and between regions (Salpastan versus 10°E) among oozoids using the Benjamini-Hochberg (BH) adjusted *p*-value < 0.001 and by setting the log fold change threshold (LFCT) to 1 in gene expression levels. Gene ontology (GO) enrichment analyses was performed for significantly up- and downregulated genes together using the R package topGo *v.* 2.44.0 and ‘*weight01*’ algorithm [19]. We filtered the GO hierarchy by having at least 10 annotated genes. Only GO terms within biological processes (BP) and a *p*-value < 0.05 (Fisher`s exact test) were considered significant.

## 3. Results

Salpastan station was characterized by a mean seawater temperature of 1.42 ± 0.50°C (range of 0.48–1.93°C) at a maximum sampling depth (200 m) and was mainly characterized by a cold water intrusion (approx. 0.5°C) between 100–200 m depth (electronic supplementary material, figure S2 and table S1). Chl *a* concentrations were exceptionally low (0.15 ± 0.03 mg m^−3^) in all water layers. At 10°E station, environmental conditions were mainly characterized by a pycnocline at approximately 100 m depth. The mean seawater temperature was 2.38 ± 0.53°C (range of 1.91–3.38°C) and the Chl *a* concentration was 0.34 ± 0.45 mg m^−3^ (range of 0.08–1.40 mg m^−3^) at the maximum sampling depth (450 m). All samples were taken during the night/just after sunrise. Total length of oozoids from Salpastan station was 17–19 mm and those from 10°E station were 22–25 mm. Blastozooids sampled at Salpastan station were 17–22 mm in length and showed a developing embryo prior to extraction. Filtering for more than 10 counts per gene among all samples resulted in 45 698 genes for downstream analyses. PCA revealed three clusters, reflecting geographical positions and reproductive forms (figure 1*b*). The regional and form effects accounted for 68% (PC1) and 11% (PC2) of the variance, respectively.

### (a) Differential gene expression analysis between reproductive forms (oozoids versus blastozooids)

About 167 genes were found to be differentially expressed (LFCT = 1, BH adjusted *p*-value < 0.001) between oozoids (*n* = 2) and blastozooids (*n* = 5) at Salpastan, of which 68.3% (114 genes) were annotated. The majority of DEG (approx. 95%) were upregulated in oozoids compared to blastozooids (electronic supplementary material, figure S3). Analysis of GO term enrichment revealed 39 enriched terms (*p* < 0.05) within BP (electronic supplementary material, table S2). Most genes (*n* = 40) were assigned to the enriched GO terms translation (40S and 60S ribosomal proteins) and processes related to muscle structure development (eight genes, e.g. troponin T, myosin heavy chain, muscle).

### (b) Differential gene expression analysis between regions (Salpastan versus 10°E)

**Figure 2.**
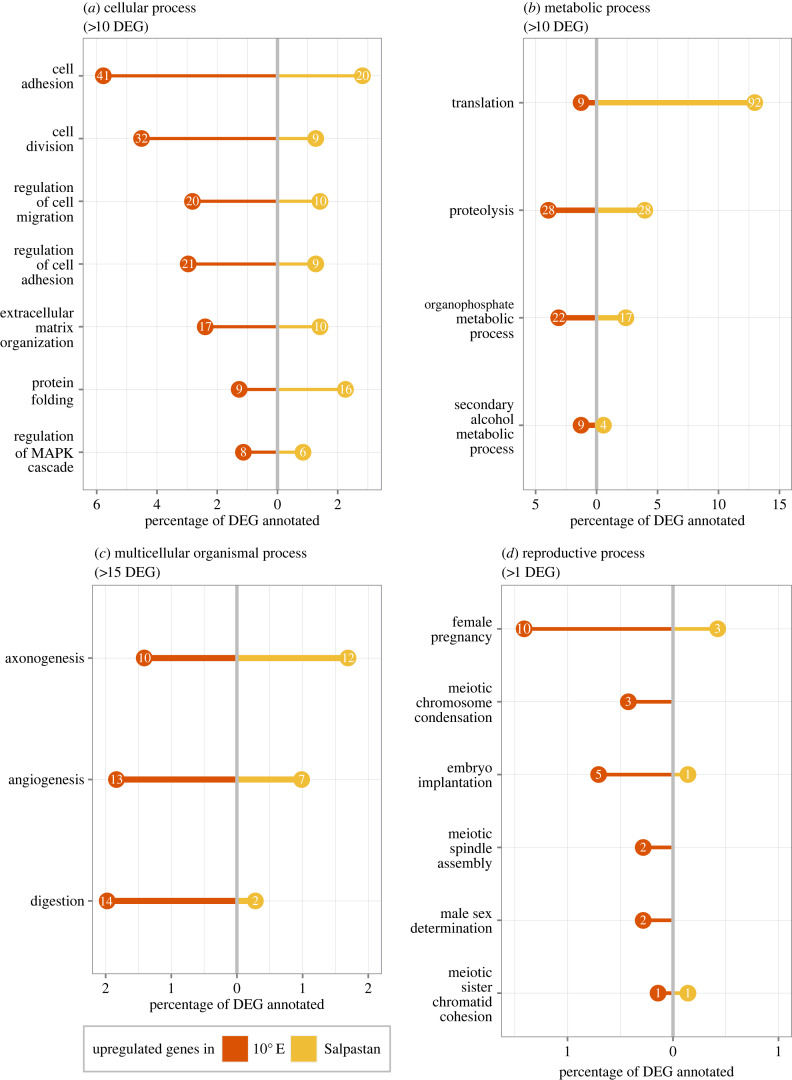
Results of GO enrichment analysis within (*a*) cellular, (*b*) metabolic, (*c*) multicellular organismal, and (*d*) reproductive processes. GO terms are filtered by coverage of DEG as indicated. See the electronic supplementary material, table S3 for a full list of all enriched GO terms within BP. Numbers in circles indicate the absolute number of genes assigned to each category, while the *x*-axis indicates the respective percentage of all annotated DEG (709, 43.7% annotation). Different colours (yellow = Salpastan, red = 10°E) indicate the different sampling stations.

DGE analysis between oozoids obtained at Salpastan (*n* = 2) and 10°E station (*n* = 3) revealed 1623 DEG (LFCT = 1, BH adjusted *p*-value < 0.001) with most genes (approx. 61%, 989 genes) being significantly upregulated in oozoids at Salpastan compared to 10°E station (electronic supplementary material, figure S4); 43.7% (709 genes) of DEG were annotated. GO enrichment analysis identified 144 enriched terms (*p* < 0.05) (electronic supplementary material, table S3). Within cellular processes, most genes were assigned to GO terms cellular adhesion (*n* = 61), and cell division (*n* = 41). Forty-one and 32 of those DEG, respectively, were upregulated at 10°E station (i.e. downregulated at Salpastan; figure 2*a*). The majority of DEG involved in metabolic processes was related to translation, covering 101 DEG (approx. 80% ribosomal proteins), of which 92 genes (91%) were upregulated in oozoids at Salpastan (figure 2*b*). Further, genes (e.g. chymotrypsinogen, trypsin-1) involved in digestion, axonogenesis and angiogenesis were differentially expressed between both regions (figure 2*c*) with digestion being the most significant process (*p* = 7.7e-07; 14 of 16 genes upregulated in oozoids of 10°E station). Within reproductive processes, 20 of 24 genes related to reproductive processes (e.g. female pregnancy) were found to be upregulated in oozoids at 10°E station compared to Salpastan (figure 2*d*; electronic supplementary material, figure S4).

## 4. Discussion

In recent decades, the abundance of salps has increased in the Southern Ocean and their distribution has shifted southwards [2,7]. However, it is still unclear whether or how salps are able to physiologically maintain their populations in the high Antarctic environment [9,11]. Here, we assessed how *S. thompsoni* copes with different environmental conditions within one season and basin. This study revealed both form-specific (oozoids versus blastozooids) and region-specific (Salpastan versus 10°E station) gene expression patterns (figure 1*b*).

### (a) Differences between reproductive forms at Salpastan station

At Salpastan, DEG analysis revealed an upregulation of processes linked to higher investment in translational capacity and active muscle development in oozoids compared to blastozooids (electronic supplementary material, figure S3 and table S2). Salpastan station was located within the persistent cold water meander and was characterized by an exceptionally low food supply (electronic supplementary material, figure S2) [13,14]. A similar difference between forms at Salpastan has previously been shown in winter samples obtained from Bransfield Strait [15], however, the upregulation of translation and increased investment in muscle development observed here probably reflects a general dissimilarity between the reproductive forms, independent of environmental conditions, owing to basic morphological differences between both forms, e.g. shape and size of muscle bands [15,20].

### (b) Response of oozoids to regionally different environmental conditions

Physiological responses to seasonally and geographically variable environmental conditions have already been studied in Antarctic species such as Antarctic krill [21,22]. However, to date the influence of environmental changes on *S. thompsoni* owing to variability between regions has rarely been studied [13]. Here, we investigated the physiological response to regionally different environmental conditions, to our knowledge for the first time. In this study, the biggest differences in gene expression patterns were observed between similarly sized oozoids sampled from the two locations (Salpastan station and 10°E station; PC1: 68%) located only approximately 1250 km apart south of the APF. The environmental conditions at 10°E station were more favourable compared to Salpastan station (electronic supplementary material, table S1 and figure S2), which may explain the strong differences in gene expression patterns observed here. Some uncertainty exists regarding the exact depth at which salp samples were collected and the environmental conditions they experienced, as samples were collected at maximum depths of 200 and 450 m at Salpastan and station 10°E respectively. However, small oozoids have been shown to migrate vertically to depths of approximately 100 m during the night [23], therefore it is likely that the small oozoids (17–25 mm) in this study were sampled near the surface, where they may have experienced the greatest differences in environmental conditions between the two stations (electronic supplementary material, table S1 and figure S2).

The distinct pattern between both regions was related to differences in the expression of genes related to metabolic, reproductive and cellular processes (figure 2). Most genes involved in cell adhesion and division were downregulated at Salpastan compared to 10°E station (figure 2*a*). The downregulation of physiological processes, such as cell division, could be an indication that energy has to be re-allocated in a cold and/or poor-food environment [24]. In addition, translation was strongly upregulated in oozoids from Salpastan station, indicating a higher demand for translational capacity under low water temperatures (figure 2*b*). This is consistent with an upregulation of genes encoding for ribosomal proteins in blastozooids during winter in the Bransfield Strait [15]. An increased expression of genes involved in ribosome biogenesis may be related to a quantitative compensation (e.g. of reduced enzyme activities) in response to low temperatures, to ensure basal maintenance in an organism [25–27], and may therefore represent certain flexibility of *S. thompsoni* to cope with the extreme environmental conditions in Antarctic regions [15,28]. Several genes related to angiogenesis and axonogenesis were also differentially expressed between oozoids from both regions (figure 2*c*). Cold-induced angiogenesis results in an increased capillary density, which may overcome the effect of impaired tissue perfusion owing to increased fluid viscosity in the cold [29,30].

In a previous study [13], distribution and population demography were investigated at the same stations (Salpastan and 10°E station). The temporal dynamics of both, seawater temperature and Chl *a* concentration (electronic supplementary material, figure S5), as well as the observed salp population development pointed towards an earlier initiation of the salp reproduction at Salpastan station [13]. Furthermore, the exceptionally high abundances observed at Salpastan station suggested a limited spawning event that started approximately 2–3 months ago prior to field sampling, successful enough to produce high salp densities subsequently reducing local phytoplankton bloom development through grazing [13]. By contrast, 10°E station showed an ongoing salp reproduction, low salp densities [13] and an increasing Chl *a* trend (electronic supplementary material, figure S5). Here, expression of genes involved in reproductive processes were downregulated in oozoids from Salpastan station (figure 2*d*), indicating that reproduction may have ceased at a certain point, probably owing to low water temperatures and decreasing food concentrations (electronic supplementary material, figure S5). This hypothesis is supported by the fact that genes related to digestion were downregulated in oozoids from Salpastan, which also reflects the low Chl *a* concentration and therefore, low food supply. Furthermore, this is consistent with an analysis of gut pigment levels, which was significantly lower in *S. thompsoni* from Salpastan compared to other regions within the APF [13].

Recent studies showed that sexual reproduction of blastozooids may suffer from unfavourable conditions [10,11]. Our analysis focused on small oozoids and therefore provides, to our knowledge, the first evidence that asexual reproduction in salps may also be affected in response to low temperature and food conditions. At Salpastan, water temperatures ranged from 0.48 to 1.93°C, roughly in line with previous findings of a proposed temperature threshold (greater than 1°C) for successful reproduction of salps [9,11,31]. However, owing to the uncertainty of the exact sampling depth, it cannot be confirmed that the generally proposed temperature threshold of greater than 1°C for successful reproduction can be applied to all circumstances and reproductive stages. Furthermore, if the observed patterns are an effect of low temperatures alone or a combination of both, the low water temperatures and very low food concentrations (approx. 0.15 mg m^−3^), remains questionable. Our findings may be in contrast to older oozoids at more advanced developmental stages, which are suggested to overwinter and prepare for reproduction at depth during winter [1,3]. The oozoids analysed here were relatively small (electronic supplementary material, table S1), indicating a more complex and possibly life stage-dependent sensitivity to environmental conditions of *S. thompsoni*.

### (c) Concluding remarks

We conducted transcriptomic analyses that were restricted to low replicate numbers (*n* = 2–5). By using DESeq2, which provides consistent performance even for small studies with few replicates [18], and applying a threshold (LFCT = 1) in DGE analysis, we increased sensitivity to true DGE signals, therefore mitigating the effect of small replicate numbers [32]. Furthermore, we only observed responses at transcriptomic level. While these were in line with salp biology data [13], responses of physiological processes may also occur at the post-translational and whole-animal level. Nevertheless, our study showed how regional differences in temperature and food supply affect the expression of genes involved in cellular processes, metabolism and reproduction in oozoids, to our knowledge for the first time. Our results therefore provide a very important insight into how *S. thompsoni* may respond to changing environmental conditions, which is crucial given the projected range shift of the Southern Ocean salp populations under climate change.

## Data Availability

Raw sequence data were obtained from Batta-Lona *et al*. [17] (Bioproject accession number PRJNA279245, https://www.ncbi.nlm.nih.gov/bioproject/?term=PRJNA279245) and Müller *et al*. [15] (Bioproject accession number PRJNA822688, https://www.ncbi.nlm.nih.gov/bioproject/?term=PRJNA822688). Detailed descriptions about the *de novo* transcriptome assembly and annotation can be found in Müller *et al*. [15]. The datasets supporting this article have been uploaded as part of the supplementary material [33].
